# Hypofractionation Radiotherapy vs. Conventional Fractionation for Breast Cancer: A Comparative Review of Toxicity

**DOI:** 10.7759/cureus.3516

**Published:** 2018-10-29

**Authors:** Ashraf Youssef, Jason Stanford

**Affiliations:** 1 Radiation Oncology, Geisinger / Holy Spirit, Mechanicsburg, USA

**Keywords:** breast cancer, hypofractionated, standard fractionation, radiation therapy, breast fibrosis

## Abstract

The use of postoperative radiation therapy after breast-conserving surgery was longstanding standard practice. The treatment protocol used a standard fractionation of 50 Gy in 25 fractions plus a boost. Recently, the hypofractionation approach has gained support based on Canadian and English studies that claimed equal tumor control and similar toxicity to the standard protocol.

We conducted a review of the literature of hypofractionation studies and compared the reported toxicity with the general literature. We placed special emphasis on breast fibrosis after hypofractionation versus standard fractionation. We found a striking difference in the breast toxicity reported by the hypofractionation literature regarding breast fibrosis as compared to standard fractionation. Breast fibrosis should be explored further via additional studies and discussed with potential breast-conserving surgery patients.

## Introduction and background

Recently, the American Society of Radiation Oncology released a task force guideline recommending hypofractionated radiotherapy for all women of any age whether they had received chemotherapy or not [1]. Their evidence-based recommendations were supported by studies from Canada and the United Kingdom [2-6]. This study is a review of the scientific and radiobiological basis for the two treatment protocols (hypofractionation and standard fractionation) and an evaluation of the short-term and long-term toxicity reported.

## Review

According to Hall, “laboratory data proved that fewer and large dose fractions result in more severe late reactions, even though the early reactions are matched by an appropriate adjustment in total dose” [7]. Hall also reports that if “a fractionated scheme is changed in clinical practice from many small doses to a few large fractions and the total dose is titrated to produce equal early effects, the treatment protocol involving a few large fractions results in more severe late effects” [7]. Fraction size, according to Hall, “is the dominant factor in determining late effects, the overall treatment time has little influence” [7].

We calculated the biological effective dose (BED) for both protocols using α/β of 3 Gy for late tissues and α/β of 10 Gy for early tissues.

In regards to the standard fractionation (50 Gy in 2-Gy fractions), the BED for late tissues was 83.3 Gy and 60 Gy for early tissues where:

BED = nd (1 + n/ (α/β) )

Comparatively, for hypofractionation (41.6 Gy in 3.2 Gy per fraction), the BED for late tissues was 86 Gy and 55 Gy for early tissues.

Table 1 presents a comparative analysis of the BED for different dose protocols.

**Table 1:** Comparative analysis of the BED for the different dose protocols BED: biological effective dose

	Standard	Hypofractionation		
BED	50 Gy in 2	41.6 Gy in 3.2 Fx	39 Gy in 3 Gy	42.56 Gy in 2.66 Gy
Early (α/β=10)	60	55	50.7	53.88
Late (α/β=3)	83.3	86	78	80
# of fractions	25	13	13	16
Average time in days	33 days	17	17	22

The UK Standardization Breast Radiotherapy START Trial A and B found that breast appearance and breast hardness were the most common changes among the two groups but the results were similar (42.6% for the 50 Gy in two fractions and 44.6% for the 41.6 Gy in 3.2 Gy per fraction or 39 Gy in 3 Gy per fraction) [2-3].

START Trial A showed no significant difference between the breast hardness caused by 50 Gy in 2-Gy fractions and 40 Gy in 15 fractions. The trial reported 38.2% of the breasts were “Harder” with hypofractionation versus 42.3% for standard fractionation.

Leong et al. found no added swelling to the arm using hypofractionation. The hypofractionation daily dose was 2.25 Gy to 2.5 Gy to a total of 45 Gy to 40 Gy, respectively. The conventional fractionation ranged between 1.8 Gy and 2 Gy to a total dose of 45 Gy to 50.4 Gy, respectively. Of 1,759 patients, 708 were evaluated (40%). The two groups were not equal, 406 patients received hypofractionation, and 302 received conventional fractionation [8].

Whelan et al. reported no difference in toxicity between 50.0 Gy in 25 fractions over a period of 35 days and a dose of 42.5 Gy in 16 fractions. Grade two subcutaneous toxicity had tripled in 10 years as compared to the 30% increase for standard fractionation [4].

Yarnold et al. found that the probability of moderate/marked breast induration after 50 Gy in 2-Gy fractions was between 39 Gy in 13 fractions and 42.9 Gy in 13 fractions with fewer side effects for the 3.3-Gy fraction size [6].

Reddy et al. compared hypofractionation versus standard fractionation for 287 women treated from 2011 to 2017 (42.56 Gy in 16 fractions plus 10 Gy to 12.5 Gy in four to five fractions boost or 50 Gy in 25 fractions plus 10 Gy to 14 Gy in five to seven fractions). The cosmetic assessment was dependent on photographs and the patient’s own assessment. There was no report of breast fibrosis. They reported similar or improved cosmetic outcomes with hypofractionation as compared to standard fractionation [9].

These studies showed a high rate of breast fibrosis with hypofractionation and a similarly high rate of breast fibrosis with standard fractionation.

Haviland reported more side effects with a fractionation schedule of 13 sessions at 3.3 Gy versus 50 sessions at 2 Gy or 39 sessions at 3 Gy. There was a statistically significantly increased rate of physician-assessed shoulder stiffness in the 42.9-Gy schedule compared to the 50-Gy treatment in the START pilot (hazard ratio, 3.07; 95% confidence interval, 1.62 to 5.83, p = 0.001). There was no such effect reported for the hypofractionated schedules in START-A and START-B [10]. Table 2 presents a comparison of the incidence of breast induration.

**Table 2:** Comparison of the incidence of breast induration

Study	Hypofractionation	Standard Fractionation 50 Gy ( 2 Gy x 25 fractions)
	Incidence of Breast Induration	
START A [2]		
41.6 Gy (3.2 Gy x13 fractions)	44.6%	42.6%
39 Gy (3 Gy x 13 fractions)	34.9%	42.6%
START B [3] 40 Gy (2.67 x 15 fractions)	38.2%	42.3%
Whelan [5] 42.5 Gy (2.65 Gy x 16 fractions) 5 years Grade 2	3.8%	5.2%
10 years Grade 2	9.4%	6.8%
Yarnold [6] (overall induration) 42.9 Gy (3.3 Gy x 13 fractions); 39 Gy (3 Gy in 13 fractions)	40.8%; 20.4%	28.6%

Xiaofeng et al. found that 50% to 60% of the patients had developed mild to moderate late breast fibrosis one year after hypofractionation radiation therapy (40 Gy plus simultaneous cavity boost to 48 Gy in 15 fractions) [11]. Yu et al. found 19% subcutaneous fibrosis after hypofractionation, yet “good” or "excellent" cosmesis was reported in 94% of the cases [12]. According to Pezner, a “course of external beam radiation therapy to the whole breast to a dose of 50 Gy in 25 fractions without local boost yields cosmetic results with little if any palpable fibrosis, even after 12 years of follow-up” [13]. Bartelink et al. reported a 1.6% incidence of breast fibrosis after 50 Gy regular fractionation and only 4.4% after adding 16 Gy boost [14]. Murphy et al. reported only 8.4% breast fibrosis (273 patients of 3,186) after using standard fractionation followed by a boost of 10 to 16 Gy. He concluded that breast fibrosis was related to large breast cup size and higher electron energy used for the boost [15]. Harris et al. reported breast fibrosis after 5000 cGy of breast radiation is rare. He also reported an increase in breast fibrosis after 6000 cGy [16].

De La Rochefordiere et al. found that using a dose of more than 2 Gy per fraction is associated with worse cosmesis [17]. Clark et al. concluded that daily fractionation with a dose of 2.5 Gy leads to greater fibrosis and breast retraction [18]. There were many reports stating that radiation oncologists in the United States are reluctant to adopt the hypofractionation regimen [19].

## Conclusions

There is some discrepancy between hypofractionation studies and many other literature reports about the incidence of breast fibrosis reported for standard fractionation. Breast fibrosis can be a potential side effect of hypofractionated radiotherapy that needs to be further studied.
